# Systematic literature review of Internet interventions across health behaviors

**DOI:** 10.1080/21642850.2014.895368

**Published:** 2014-04-22

**Authors:** Su-I Hou, Su-Anne Robyn Charlery, Kiersten Roberson

**Affiliations:** ^a^ Health Promotion and Behavior, University of Georgia, 309 Ramsey Center, 330 River Road, Athens, GA30602, USA

**Keywords:** Internet, intervention, health behavior, review

## Abstract

*Purpose*: This review examines Internet interventions aiming to change health behaviors in the general population. *Methods*: Internet health interventions in the USA published between January 2005 and December 2013 were identified through Medline and CINAHL. Keywords used were (*Internet or e-health or social media or web*) paired with (intervention or program*)*.* A total of 38 articles met all criteria and were reviewed. *Results*: Studies were analyzed by targeted health behavior interventions: tobacco (5), alcohol (4), weight loss (7), physical activity (PA) (7), nutrition (2), PA and nutrition combined (5), HIV or sexual health (4), and chronic diseases (4). Interventions ranged from one session to 24  weeks (average 6–12 weeks). Common strategies used, including web-based information, tailored feedback, weekly e-mails, goal setting, and self-assessment. Social cognitive theory and the transtheoretical models were the most commonly used frameworks. Recruitment strategies were typically media based varied by settings and populations. Except for the tobacco interventions, the majority studies yielded significant outcomes. *Conclusion*: This review provides updates and synthesized knowledge on the design and consistent effectiveness of Internet interventions across health behaviors. Results have implications for public health and healthcare professionals, as they play a key role in developing and delivering health promotion interventions as well as in assisting the communities and clients serviced obtaining evidence-based health information.

## 1. Introduction

The Internet is a powerful global communication medium that provides instantaneous information across geographical, cultural, language, and time spheres. Increased Internet access worldwide has resulted in a great number of health researchers and practitioners using it as a platform for the delivery of public health interventions. In recent years, several research studies have evaluated Internet health interventions and their intention to change health behaviors in the general public. Although there has been limited research to determine the efficacy of health-related information on the Internet, a systematic review of the early years of scientific evidence using e-health interventions, before 2003 and during 2002–2009, indicates significant positive effects on health behaviors in diverse populations and across a wide range of health conditions (Neuhauser & Kreps, 2010; Risk & Dzenowagis, 2001).

Most of the existing Internet study reviews focus on specific topical issues, with the vast majority focusing on nutrition, dietary, and physical activity (PA) behaviors, and on weight-loss interventions (Hamel, Robbins, & Wilbur, 2011; Neuhauser & Kreps, 2010). Current reviews on Internet health interventions also tend to focus on specific components, such as the evaluation of the delivery methods, dose of treatment, attrition and/or user satisfaction, rather than providing a more comprehensive review on the interventions' integrated components in their findings. The limited integration of intervention components in findings reported in existing studies can make it difficult to interpret results or determine what characteristics or components of the Internet health interventions are the key to achieving behavioral changes in measured outcomes (Hamel et al., 2011). It is necessary that studies on Internet health interventions analyze the existing literature in a way to better understand and improve the feasibility, usability, and efficacy of such interventions (Risk & Dzenowagis, 2001).

An examination of Internet health intervention studies on a variety of topics and their existing components allows for comparisons and contrasts across a variety of health conditions or outcomes. Evaluating e-health interventions from this perspective could provide new insights for researchers and practitioners to better develop, adapt, or adopt Internet-based health interventions. To the best of our knowledge, there is currently no review on Internet health intervention studies conducted in the USA that examines across age, gender, and health conditions, without focusing on a specific intervention component or measurement outcome. The insights gained from this literature review that crosses demographic categories will provide a deeper understanding for effective Internet health intervention development.

For the purposes of this study, the term Internet health intervention was operationally defined as systematic intervention programs, usually addressing one or more determinants of health delivered primarily via the Internet and interfacing with an end user (Bennett & Galsgow, 2009). We included only behavioral health interventions where the major component was delivered via the Internet to distinguish its effects from other interventions provided primarily by other media, such as print material, CD-ROM, videos, and phones (MMS and SMS). This review focuses primarily on the effectiveness of Internet health interventions, employing a more comprehensive approach by not limiting the scope of the research to a particular health condition. It also evaluates a multitude of considerations for using the Internet as a platform of health intervention development and delivery, such as their sampling and recruitment strategies, target populations, theoretical framework or models for behavior change, program delivery, and outcomes of the interventions. Throughout this review we compared and contrasted strategies used by various Internet health interventions, identified trends and themes as they emerge, and discussed the value of using behavior change theoretical frameworks, recruitment strategies, differences in target populations and settings, and any enhancements associated with supplemental strategies as they relate to the health behavior or outcomes.

The aim of our study is to provide a comprehensive narrative review of the literature published on Internet health interventions between January 2005 and December 2013 and to identify patterns from the studies reviewed as well as bridge the gap of current knowledge to inform the development of future research. We provide a detailed analysis from a recent sample of Internet health interventions to examine intervention characteristics, determine the evidence of their effectiveness as well as identify strategies to improve health outcomes.

## 2. Methods

Medline and CINAHL, two major electronic databases, were used to locate intervention studies that used computer-based information and communication technology to deliver a specific health intervention. The key search terms (*Internet or e-health or social media or web*) were paired with the search terms (intervention or program*) in the title field*.* Internet health intervention studies in the USA published between January 2005 and December 2013 were included for the purpose of this review. This time period were decided to reflect a reasonable time-period coverage during the rapidly changing Internet era to provide a timely update while not duplicating existing review efforts as well as aimed to paint a good picture of the current state of Internet health studies. The last search for this systematic review was performed on 15 January 2014.

### 2.1. Study selection

Inclusion criteria were original research conducted in the USA, published in English between January 2005 and December 2013, and contained the above search-terms in the title. Further inclusion criteria included research design using at least one comparison or control group (CG), network provided by the Internet during the studies' intervention or program, use of a web-based computer program as a significant part of the intervention, and health behavior-related outcome(s) as a primary part of the evaluation. The bibliographies of retrieved articles were also scanned for additional references.

### 2.2. Data extraction

The following information was systematically extracted by two reviewers; author/s and year of publication, target group and demographics, recruitment methods, targeted health behaviors, setting or location, theoretical frameworks, research design and follow-up, characteristics of the intervention (intervention type/components and delivery strategies), and effectiveness of the study. During the process of completing the final review, the information was compared and contrasted, which can be found in Tables 1 and 2

**Table 1.** Recruitment methods and intervention strategies of internet intervention studies reviewed.

Study (author/s, year)	Target group demographics and size (*n*)	Recruitment methods	Setting/location	Study design/intervention
*Tobacco prevention*				
Japuntich et al. (2006)	*N* = 284 smokers	Billboards, bus interior posters, flyers, television ads and press releases	Milwaukee and Madison, WI	RCT; 12 weeks; CHESS smoking cessation and relapse prevention – 20 minutes individual counseling to increase motivation; bupropion 2×/day, follow-up, study computer, dial-up Internet connection and 12 weeks of access to CHESS SCRP website. They were encouraged to access once per day. Phone calls received if no log in for one week
McKay, Danaher, Seeley, Lichtenstein, and Gau (2008)	*N* = 2318; 18+ year-old current smokers – wanting to quit within next 30 days	Internet-based recruitment – Google and Yahoo ads, and word of mouth	N/A	RCT; smoking cessation program using models, including cognitive behavioral strategies, smoking cessation info, behavior self-management, behavior change recommendations, peer support, and engaging video testimonials
Patten et al. (2006)	*N* = 139 adolescent smokers 11–18 years	Television commercials, radio and newspaper announcements, flyers displayed in schools and clinics; incentives of $10 for each assessment visit at weeks 4–24 and $20 at week 36	Rochester, MN; Madison, WI; Hartford, CT	RCT; access to “Stomp out Smokes” and Internet for 24 weeks, 38 components; services for private journaling service, quizzes, quit plan and quit notes, and art gallery; assessment visits staff had no personal contact with participants; no telephone or email prompts provided
Severson et al. (2008)	*N* = 2523 smokeless tobacco users	News releases to print and broadcasting media, paid ads on Google.com, links on other websites, paid ads in newspapers and magazines, direct mailing to identified smokeless tobacco users, targeted mailings to health care and tobacco control professionals; incentives – $10.00 for each follow-up assessment and $20 if all three completed	USA or Canada	RCT; personal quitting assistant, streaming video (displaying quitting info and testimonials), streaming video, and broader range of printable resources, “ask and expert” forum and peer forum, and links to other websites; up to three tailored emails before their quit date, supportive message sent 1, 7, and 14 days after their reported quit date and three re-engagement emails sent 7, 30, and 60 days after last login
Swartz, Noell, Schroeder, and Ary (2006)	*N* = 351; 18+ year-old current smokers – wanting to quit within next 30 days	Recruited through large worksites, promotional materials w/ www.Quitcigs.org address, employees placed Quitcigs website on intranet sites or in emails and electronic newsletters to employees	Large worksites	RCT; web program automated approximation of live smoking cessation counselor, five major content modules addressing benefits, barriers, avoidance and craving strategies, and development of a “Quit w/n 30 days Calendar”
*Alcohol prevention*				
Bingham et al. (2010)	*N* = 1137 first-year college students, 18–20 years	Random selection by email invitations to students' unique address, flyers in dorms and campus shuttles, student testimonial and endorsement by Student Health Services Director; incentives: baseline = $10, post-test = $15, sessions 1–3 = $10 ea., session 4 = $15 and mid-interval survey = $10	University of Michigan	Intervention/control design; nine weeks M-PASS: four 10–15 minutes online sessions tailored to participants’ alcohol-related risk, incorporated interactive activities, generated feedback on topics, tailored and reminder emails sent throughout intervention to guide participants to completion. Activities – quizzes, self-assessments, and exercises w/ avatars
Doumas and Hannah (2008)	*N* = 124 young adult, 18–24 employees	Recruited through five HR departments in local companies with high numbers of employees in 18–24-years age group	Metropolitan, North West workplaces	RCT; web-based intervention (WI) – brief program providing personalized normative feedback about drinking. Individual graphed feedback showing levels of drinking, summary of numbers of days individual drank and approximate financial cost of drinking in the past year. WI with motivational interview – in addition to website-15 minutes in person motivational interview
Moore, Soderquist, and Werch (2005)	*N* = 116 college students (18–25 years)	Convenience sample from three college courses	South Eastern US university	RCT; four weekly newsletters identical in appearance for web and print versions; five main components; question challenging alcohol-expectancy belief, definitions of a standard drink and binge drinking; web version provided links to alcohol-related info, services at the university, and other informational and interactive alcohol websites
Neighbors, Lee, Lewis, Fossos, and Walter (2009)	*N* = 295 college students, 21 +	Email and postal mail invitations	Two Northwestern universities	RCT; two days and day before 21st birthday – email sent w/ link to a birthday card w/ humorous msgs about moderation on 21st birthdays; personalized feedback about drinking intentions and expectations for their upcoming 21st birthday; printable BAC chart based on gender and weight; protective behavior strategy
*Weight loss*				
Gold, Burke, Pintauro, Buzzell, and Harvey-Perino (2007)	*N* = 124 overweight and obese adults 18 years and older	Advertisements in local newspaper	Burlington, VT	RCT; VTrim six month online therapist-led weight maintenance program; specific behavior modification lessons featured ea. week; self-reported weight ea. week; hr long weekly online chat-trained therapist; weekly feedback to homework emailed-trained therapist; online journal used to track energy intake and feedback on entries-therapists
Jones et al. (2008)	*N* = 105 male and female high school students	Flyers and presentation in health education and physical education classes, student rallies, and parents back to school night	Boise, ID and Hayward, CA	RCT; StudentBodies2-BED 16 week; weekly new topic on healthy eating, PA, binge eating and weight loss/maintenance; interactive components – self-monitoring journals for dietary intake, PA weight and person thoughts. Group discussion w/ research assistant; parents handbook; weekly letters and motivational msgs
Rothert et al. (2006)	*N* = 2862 overweight and obese persons (BMI 27–40 kg/m^2^)	Kaiser members informed by clinicians, member newsletters, flyers, and letters sent to those in diabetes and CVD registries	Kaiser Permanente Regions Georgia, Mid-Atlantic States, Northwest and Ohio (health care setting)	RCT; tailored expert system condition used balance – 6 week self-help program; opportunity to enroll “buddy” who were sent email messages and encouraged to provide informal support; materials consisted of an initial guide followed by tailored action plan delivered at 1, 3, and 6 weeks into prog.
Tate, Jackvony, and Wing (2006)	*N* = 192 obese or overweight adults	Advertisements in local newspapers; incentives − $25.00 and $50.00 paid for three- and six-months follow-up	N/A	RCT; one face-to face session w/ behavioral weight-loss recommendations for diet, exercise and behavior changes; meal replacements for first week and coupon given to offset cost; website access to electronic diary and message board; weekly feedback from preprogrammed computer or via email from weight-loss counselor in person
Webber, Tate, and Bowling (2008)	*N* = 66 women with BMI between 25 and 40	Newspaper ads, telephone screening, and information session; incentives − $40 for attending follow-up session	N/A	RCT; one face-to-face session, calorie book and self-monitoring diaries; 16 week behavioral weight-loss prog. w/ weekly lessons and web links to tips and related sites. Enhanced group had weekly one hour online chat group sessions led by a doctoral student
Williamson et al. (2005)	*N* = 57 overweight AA girls with at least one obese parent	Media and advertising campaign, including talks in community	N/A	RCT, culturally specific, counselor-conducted four face-to-face sessions with parent and adolescent, online correspondence w/ both arms. IG – behavioral treatment and contracting. CG – received nutrition education
Turner-McGrievy and Tate (2013)	*N* = 96 overweight and obese men and women	Listservs, television advertisements; participants received a $20 incentive for completing all three parts and an additional $20 for the completion of the six-month assessment activities	N/A	RCT; participants were assigned to each group; each group received podcasts (15-minutes two times/week for three months; five-minutes two times/week for 3–6 months); the IG was required to download a PA monitoring application and Twitter to their phone and log on daily; a weight-loss counselor posted two messages a day to reinforce the podcasts and stimulate discussions
*PA and nutrition*				
Bosak, Yates, and Pozehl (2009)	*N* = 22 metabolic syndrome adults	Enrolled active patients in university cardiology lipid clinic database	N/A	Two-group pre- and post-test; six weeks w/ instruction and feedback – four sources of efficacy info; multi-component self-efficacy strategies: education, behavior-specific goal-setting and self-efficacy strategies; knowledge tests, new content on website each week, including links for evidence-based websites, daily entry of PA, tips 3×/week, goals reviewed weekly
Carr et al. (2008)	*N* = 32 men and women, 21–65 years	Passive recruitment via advertisements and email solicitations from predominantly rural regions	Wyoming and Northern Colorado	RCT; ALED-I 16 week internet prog. supervised by licensed program admin, w/ copy of complementary workbook, self-paced prog., behavior modification issued, “check in” to review and complete “Ready Set Go” lessons, monitor sedentary activities, substitute alternatives and match with virtual participants with same level of readiness to change, completion of “my journal” and five-question quiz
Cavallo et al. (2012)	*N* = 134 female undergraduate students	Participants were directed to an online screener via print and electronic communications (email, Facebook and Twitter); $30 incentive for completing all study parts	Large southeastern public university, University of North Carolina at Chapel Hill	RCT; online social networking and self-monitoring of PA; INSHAPE website and a Facebook group; a moderator was used to encourage participation and to answer questions from participants
Dunton and Robertson (2008)	*N* = 156 ethnically diverse adult females	Posters and fliers used at local health centers and clinics; $25 for completing surveys	Northern and Southern California	Randomized trial; Women's Fitness Planner-produced individualized PA feedback; 10 weekly emails with links to web page with an interactive tailoring tool to promote PA
Irvine, Gelatt, Seeley, Macfarlane, and Gau (2013)	*N* = 368 sedentary men and women (mean age 60)	A mixture of online recruitment strategies (listservs, advertising on a website for seniors), flyers, newsletters and announcements to senior service agencies	Online PA intervention; N/A	RCT; weekly 10–15 minutes sessions for 12 weeks. Active After 55: text and video messages integrated with interactive values clarification and goal-setting activities. Include personal activity plan, the health value exercise, overcome obstacles, track progress, stay motivated, safety tips, tip sheets, etc. A narrator and personal coach presented video-based education content

Marcus et al. (2007)	*N* = 249 healthy sedentary (< 90 minutes PA/week) adults	Primarily newspaper ads; $10 monthly	Providence and Pittsburgh	Two-site – three arms randomized trial – (1) Motivationally tailored Internet: email prompts, daily online logging. (2) Motivationally tailored print: mailed prompts, pencil and paper logs calendar logs. (3) Standard Internet: PA logs at same intervals as other two groups
Ornes and Ransdell (2007)	*N* = 112 college aged women	Newspaper ads, posters and researcher visits to college classrooms	Public university in the South west	RCT; four weeks; delivered through WebCT with nine modules, including using pedometer, monitoring progress, recording steps; several identifying barriers and strategies to overcome them; links to maps and incorporated state supported PA site
Cook, Billings, Hersch, Back, and Hendrickson (2007)	*N* = 419 employees of a human resources company	Email letter from management, posters in offices; incentive $50.00 and $500.00 raffle prize	Atlanta, GA; Minneapolis, MN; Fountain Valley, CA	RCT; web-based group and print group. Health connections multimedia program on nutrition/weight management, stress reduction and fitness/ PA
Franko et al. (2008)	*N* = 476 university students, 18–24 years	Sign-up tables in university high-traffic areas	Northeastern University, College of Charleston, Florida Atlantic University, University of Missouri – St Louis and Columbia campuses, Florida International University	Randomized trial; MyStudentBody – three info links – (1) (Ask the Expert, student voices, College News). (2) Rate Myself assessment. (3) Main topic pages (Nutrition 101, Eating on the run, weighing in) first web session = 45 minutes (first meeting). Two weeks later − 1.5 hour meeting, second 45 minute web session completed. Three weeks after post-test EG II remotely log onto website for ∼45 minutes and visited main topic pages-text-based and audio information, interactive activities and goal-setting areas
Frenn et al. (2005)	*N* = 178 seventh graders	Requested participation from students in six classes	Midwestern urban public school	Quasi-experimental; eight session blackboard delivered, with four 2–3 minute videos used in science class used to raise awareness and consciousness of certain healthy behavior; computer-generated tailored feedback for PA and dietary fat; workbook for recording notes and evaluating sessions and Internet sites; each session lasted a class period
Thompson et al. (2008)	*N* = 80 (8–10-year-old AA girls)	Broadcast and non-broadcast methods; weekly incentive of $5	Houston, TX	RCT; eight week use of website with role modeling, comics, problem-solving and goal setting. Data collection occurred on website
Winnett et al. (2007)	*N* = 1071, 14 churches	Pulpit announcements, flyers, posters, church bulletins and “kick off” luncheons; Incentives – 20.00 at pre-test, 30.00 at post-test, and $40.00 at follow-up	Baptist or South Methodist Churches in the Southwest Virginia	GRT; GTH – 12 weekly modules targeting decreasing dietary fat, increasing fiber and FV. Support for IG from pulpits and church bulletins
Milan and White (2010)	*N* = 408 female college students, 18–29 years old	Advertisement of online nutritional study w/ monetary incentive through university email system; incentive = $30	University of Maine	GRT; stage-tailored education on folic acid MV intake for six weeks. Five education modules corresponding to stage of readiness for meeting folic acid intake recommended – four web pages each; four tailored email msgs (1/week)
Thompson et al. (2009)	*N* = 473 boy scouts (10–14-year-olds	Presentations to Houston Boy Scouts of America and troop leaders	Houston, TX	RCT; “5-a-Day Achievement Badge program”: 55 minutes of 9 weekly programming led by trained staff 30 minutes (in troop) and 25 minutes Internet. In-troop recipe preparation and tasting, recipe booklet; website used to set and report goals and goal-attainment; comic characters; problem-solving poll
*HIV and chronic diseases*				
Bowen, Keith, and Mark (2007)	*N* = 90 MSM who live in a rural area	Face-to-face, Internet banners; gift certificates of ($10, $15, and $20 given after each of three assessments)	N/A	RCT; two modules include a conversation between an HIV-negative man and an HIV-positive man with interactive graphics
Bull, Vallejos, Levine, and Ortiz (2008)	*N* = 2623 online, *N* = 1444 clinic-based, 18–24 years	Posted 3.5 million banner ads online and clinics; incentives $25–$35 upon completion	Clinic sample Colorado Internet sample, 18–24-year-olds	Quasi-experimental; stories 60–90 second-long delivered with voice and music; pictures matched to gender, race/ethnicity; participants respond to HIV risk-related questions in five modules
Carpenter, Stoner, Mikko, Dhanak, and Parson (2009)	*N* = 112 MSM, 18–39 years old, engaged in unprotected sex w/ a man w/n the last three months	Banner advertisement posted on same-sex community websites, profiles of the study listed on three popular social networking websites commonly visited by minorities; incentives $35 after tutorials; $50 after post-test	N/A	Randomized; intervention website – seven motivational, informational and skills training modules. Interactive assessment of HIV risk factors, w/ targeted feedback, mini-assessments gauging readiness to change risky behaviors, motivational exercises and communication skills training
Bull, Levine, Black, Schmiege, and Santelli (2012)	*N* = 1578 AA and Hispanic/Latino youth	Research assistants were approached directly or waited for people to approach a table; recruited online; ads were submitted to newspapers in geographic areas with high rates of STIs; recruited participants then were incentivized to recruit up to three friends	Denver, CO and a college community in Louisiana	Cluster RCT; participants were randomly assigned to the intervention or CG; “18–24 News” was the control Facebook page that shared interesting news; “Just/Us” was the intervention Facebook page that was developed to deliver information on sexuality; facilitators would update the pages daily
Bond et al. (2007)	*N* = 62 adults, 60 years and older with diabetes	University of Washington Diabetes Center, Puget sound health system and local diabetes fairs	Seattle	RCT; website emphasizing goal-setting and problem-solving skills were emphasized. Disease management, diet and exercise. CG received standard diabetes care
Chan and Vernon (2008)	*N* = 97, aged 49 and older	Clinic site recruitment during clinic visit over consecutive 19 month period; incentive for completion was $55.00	Texas	RCT; NetLET: personalized e-mail from primary care physician as reminder for screening and last test result, link to secure web page w/ more information about CRCS, video decision aid about CRCS and FOBT and links to websites about CRCS from CDC, the Mayo Clinic and Medline Plus. FOBT kit was mailed to group
Joseph et al. (2007)	*N* = 314 urban AA 9th to 11th grade students	Informed all 9th to 11th grade students’ caregivers about respiratory health survey administration in English class	Six public high schools: Detroit	RCT; Puff City – four consecutive educational computer sessions that make use of normative and positive feedback (compared with last session); voiced over msgs to accommodate low literacy
Smith, Egbert, Dellman-Jenkins, and Nanna (2012)	*N* = 38 care givers and stroke survivors from the USA	Put out national notices on websites and listserv announcements for key organizations	Nationally in the USA	RCT; IG: five components that were designed to provide care givers with knowledge, resources, and skills to help them reduce their distress and provide the best care to the stroke survivors: control: had access to the Resource Room only, was able to watch one video that described the Resource Room; provided a weekly care giving tip online

Notes: GRT, group randomized trial; prog., program; MV, multivitamin; BAC, blood alcohol concentration; CHESS SCRP, comprehensive health enhancement support system for smoking cessation and relapse prevention; M-PASS, Michigan prevention and alcohol safety for students; BMI, body mass index; ALED-I, active living every day; GTH, guide to health; STIs, sexually transmitted infections; CRCS, colon cancer screenings.

**Table 2.** Theoretical frameworks and program effectiveness of Internet intervention studies reviewed.

Study (author/s, year)	Target health issues/behaviors	Theoretical frameworks	Effectiveness (outcome evaluation)
*Tobacco prevention*			
Japuntich et al. (2006)	Smoking cessation	NA	Access to CHESS (smoking cessation support website) was not significantly related to abstinence at the end of the treatment period, or at six months post quit. The # of times participants used CHESS was related to abstinence at both end of treatment and at six-month follow-up (OR = 1.79, 95% CI = [1.25–2.56]; OR = 1.59, 95% CI = [1.06–2.38])
McKay et al. (2008)	Smoking cessation	SCT	No differences between intervention and control at three- and six-month assessments for smoking abstinence, predictors of smoking abstinence, and participant program exposure
Patten et al. (2006)	Smoking cessation	SCT	No sig. differences in 30-day, 24-week or 36-week abstinence rates for IG and CG. IG was associated with sig. greater reduction in avg. no. of days smoked than CG (*p* < .01)
Severson et al. (2008)	Smokeless tobacco cessation	NA	Sig. higher quit-rates in enhanced than basic condition.Abstinence = 40.6% in enhanced condition (IG) vs. 21.2% basic condition (CG); *p* < .001
Swartz et al. (2006)	Smoking cessation	NA	At 90 days, higher cessation rate among IG (24.1%) than CG (8.2%); *p* = .002
*Alcohol prevention*			
Bingham et al. (2010)	Alcohol-use attitudes, beliefs, and risk behaviors	Principles of MI and a model based on the HBM, TPB, TTM and PAPM	Sig. differences in attitudes and beliefs observed for women than men. High-risk IG women had sig. lower tolerance of drinking than CG; high-risk IG men had lower quantity of drinking and less frequent binge drinking than CG; low-risk IG women had lower quantity of drinking per occasion compared to CG
Doumas and Hannah (2008)	Alcohol use	Motivational enhancement model and social norming	At 30 day follow-up two IGs combined (MI and web-based feedback – WI combined) had sig. lower level of drinking than the CG for weekend drinking, frequency of drinking to intoxication and peak consumption. No differences between WI and MI interviewing arms
Moore et al. (2005)	Binge drinking	Extended parallel process model	No significant difference between the two intervention methods. Internet-based binge drinking program was feasible based on accessibility, convenience, ease of use, lower cost, higher process eval. response rate, and favorable participant feedback
Neighbors et al. (2009)	Reduced 21st birthday drinking	NA	IG associated with lower BAC than CG at step 1 (*d* = 0.33)
*Weight loss*			
Gold et al. (2007)	Weight loss	NA	VTrim (IG) lost more weight than eDiets.com (CG) at six months; (8.3 *±* 7.9 kg vs. 4.1 ± 6.2 kg; *p* = .004). At 12 months VTrim maintained greater weight loss (7.8 *±* 7.5 kg vs. 3.4 ± 5.8 kg; *p* = .002). VTrim also had higher social support scores
Jones et al. (2008)	Binge eating and overweight	NA	At completion, 27% of IG were not at risk for being overweight compared with 12% in wait-list control (WLC) group (*x*^2^ = 3.4; *p* = .067). IG had greater BMI score changes to WLC group; *p* < .01, greater reductions in objective and subjective binge episodes, and reduced weight and shape concerns
Rothert et al. (2006)	Weight loss	NA	IG lost sig. more weight than CG (3 kg ± 0.3% vs. 1.2 kg ± 0.4%; *p* < .001)
Tate et al. (2006)	Weight loss	NA	Sig. greater weight loss in computer-automated feedback (−5.3 ± 4.2 kg) and human email counseling (−6.1 ± 3.9 kg) than no-counseling group (−2.8 ± 3.5 kg) at three months; *p* < .05. At six months human email counseling group had sig. greater weight loss
Webber et al. (2008)	Weight loss	MI	Both groups lost weight over time (*p* < .001). Minimal group (access to weight-loss website) lost 5.22 *±* 4.46 kg and enhanced group (identical to minimal + online chat sessions) lost 3.71 *±* 4.46 kg. Greater number of website visits associated with greater weight loss in both groups *p* < .01
Williamson et al. (2005)	BMI, body composition, dietary intake, weight loss, and weight-loss behavior	NA	IG lost more body fat in adolescents (1.6%) and parents lost more body weight (group difference = 2.1 kg) compared with CG. Dietary fat intake was lowered for both groups in IG
Turner-McGrievy and Tate (2013)	Weight loss	SCT	No differences in percent weight loss between intervention or CG at three months (−2.6 ± 3.8% – podcast vs. −2.6 ± 3.5% – podcast and mobile; *p* > .05 between groups) or six months (−2.7 ± 5.1% – podcast vs. −2.7 ± 5.6% – podcast and mobile; *p* > .05 between groups); twitter posts decreased between the 0–3 months period to the 3–6 months period; adjusting for demographic variables Twitter was a good predictor of % weight loss at six months, each post correlated with −0.5% weight loss
*PA and nutrition*			
Bosak et al. (2009)	PA	Bandura self-efficacy	Sig. improvements found in IG HDL cholesterol (*z* = −2.024; *p* = .04) and self-efficacy for PA (*z* = −1.970; *p* = .04). Intervention appears feasible: IG consistently accessed website
Carr et al. (2008)	PA	TTM and SCT	Increased PA in IG by ∼1384 steps/day; (*p* = .03) vs. ∼816 steps in CG (*p* = .14). Decreased waist circumference and coronary risk ratio in IG and no change in CG
Cavallo et al. (2012)	PA	NA	Sixty-four of the IG participants (96%) joined the Facebook group; during the intervention website logins and Facebook activity decreased; 63% (56) of intervention participants who completed the post-study reported logging onto the Facebook group 2–3 times/month; 66% of participants indicated that they would recommend the program to friends
Dunton and Robertson (2008)	PA	HBM and TTM	After three months walking increased at faster rate in IG vs. CG: *B* = 15.04 (SE = 8.38), *p* = .035; sig. group difference in the rate of change in moderate-to-vigorous PA *B* = 17.02 (SE = 10.11), *p* = .045
Irvine et al. (2013)	PA	TPB	Sig. improvement on 13 of 14 outcome measures compared to the CG at post-test (*p* < .001; large effect size). At six months, intervention participants maintained large gains on all 14 outcome measures
Marcus et al. (2007)	PA	TTM and SCT	Baseline – six months and baseline – 12 months change scores for three arms = NS six months: print = 112.5 PA minutes/week, tailored internet = 120, and standard Internet = 90 minutes; *p* = .15 12 months: 90, 90, and 80 minutes/week, respectively (*p* = .74)
Ornes and Ransdell (2007)	PA	N/A	IG increased their mean steps/day by 38.8%, while CG increased their mean steps/day by only 2.1% [*F* (1) = 2.61, *p* = .001]
Cook et al. (2007)	PA and dietary practices, stress	SCT	IG had significantly higher scores on attitudes toward a healthful diet (*F*_1415_ = 7.104, *p* *=* 0.008) and dietary stage of change (*F*_1408_ = 6.487, *p* = 0.01). Both group showed improvement on most dietary measures. No sig. differences found between groups for stress or PA
Franko et al. (2008)	PA and nutrition	NA	IG 1 and 2 increased fruit and vegetable (FV) intake by 0.33 and 0.24 servings, respectively. Sig. increase in motivation to change eating behaviors (*p* < .05), increase social support and self-efficacy for dietary change (*p*'s < .05), and improve attitude toward exercise (*p* < .05)
Frenn et al. (2005)	PA and dietary fat	Health promotion/TTM	IG who completed > ½ sessions increased exercise by 22 minutes; *t*(103) = −1.99, *p* = .05 compared to a decrease of 46 minute for CG, and decreased the percentage of dietary fat from 30.7 to 29.9; *t*(87) = 2.73, *p* = .08. IG who completed all three sessions increased activity by 33 minutes
Thompson et al. (2008)	FJV consumption, PA and self-efficacy	SCT elaboration likelihood model	Sig. pre-to-post differences in FJV consumption (*p* = .002), PA – yesterday (*p* = .001), PA – usually (*p* = .001), and FJV self-efficacy (*p* = .003)
Winnet et al. (2007)	Nutrition and PA	SCT	Increased FV at post-test IG (∼1.5 servings) vs. CG (∼0.5 servings); *p* = .005, increased fiber 3.0 g vs. 1.5 g; *p* = .006, increased steps daily 1500 vs. 400 steps; *p* = .05, decreased weight ∼−0.3 kg vs. +0.6 kg; *p* = .03
Milan and White (2010)	Nutrition – folic acid intake	TTM	Sig. greater proportion of college women in IG-stage tailored intervention (32.6%) vs. CG non-tailored education (19.9%) taking MV @ post-test than pre-test; *p* = 0.015
Thompson et al. (2009)	Fruit juice (FJ) and low-fat vegetable consumption	SCT	Significant increases in FJ consumption (*p* = .003), FJ home availability (*p* = .009), and low-fat vegetable consumption self-efficacy (*p* = .004) among IG immediately, but not maintained six months later
*HIV and chronic diseases*			
Bowen et al. (2007)	HIV risk reduction in MSM	SCT	HIV AIDS knowledge, self-efficacy and outcome expectancies increased after participating in the intervention and changes were maintained at one week follow-up
Bull et al. (2008)	Promotion of condom use	Constructs: attitudes, norms, awareness of HIV risk, self-efficacy	Condom use: no change in clinic sample. Slight increase in condom norms in IG
Carpenter et al. (2009)	HIV and sexual risk behavior reduction in MSMs	IMB theory of HIV risk reduction and several themes and strategies from MI	# of unprotected acts decreased from baseline to follow-up for both IG and CG for all sexual practices. # of unprotected acts with risky partners decreased more for IG than CG for all types of anal and oral intercourse except receptive anal intercourse
Bull et al. (2012)	Sexual health	NA	828 of the initial 1017 were identified for participation and 652 (control = 312; intervention = 340) agreed to participate (controls recruited 1.04 on average for 636 controls and intervention recruited 1.79 on average for 942 participants); 69% of intervention and CGs completed the two-month follow-up; retention dropped to 59% at six-months for controls and 45% at six-months for interventions (a total of 75% completed follow-up); the simple effects evaluation analysis showed that at two-months there was a difference in control/IGs – condom use remained stable in the IG and declined in the CG
Bond et al. (2007)	Diabetes management, hemoglobin A1c, blood pressure, weight loss, total cholesterol, and HDL	NA	Reductions in HbA1c, weight and cholesterol level, and significant improvement in HDL levels in the IG vs. CG (*p* = .001)
Chan and Vernon (2008)	Colon cancer screening	NA	Viewing of NetLET colorectal cancer screening electronic intervention: public Internet access group = 1 of 11, private Internet access group = 10 of 42; returned FOBT kit: 11 of 42 (26%) private access IG and 8 of 35 (23%) private access CG (received physician reminder letter), no public access IG, and 3 out of 9 public access CG
Joseph et al. (2007)	Asthma management	TTM and HBM	At 12 months IG students reported, fewer symptom-days and nights, schooldays missed, restricted activity days and hospitalizations compared with CG; adjusted relative risk and 95% CI were 0.5 (0.4–0.8); 0.4 (0.2–0.8); 0.3 (0.1–0.7); 0.5 (0.3–0.8); and 0.2 (0.2–0.9), respectively (*p* < .05)
Smith et al. (2012)	Depression, stroke survivors and their care givers	NA	Findings suggest that the intervention reduced the outcome of depression for the care givers at statistically significant level (40% of CGs in the IG reported a 50% decrease from baseline scores); depression was not reduced for stroke survivors at significant levels they did show a change in the predicted direction (depression reduction did not appear until one month after treatment)

Notes: NS, non-significant/no significant; sig., significant; OR, odds ratio; CI, confidence interval; HDL, high density lipoprotein; SE, standard error; IMB, information-motivation-behavioral.

## 3. Results

**Figure 1.**
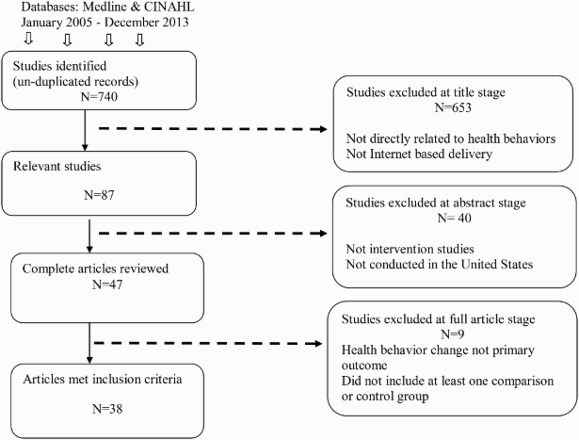
Internet health intervention review flow chart.

Literature searches yielded 913 studies. After duplicate records were removed, 740 unique records remained. Two reviewers examined all retrieved journal articles using the above criteria to determine relevance for inclusion. Titles and abstracts of these studies were screened and 693 were excluded on the basis that they were not directly Internet-based, not intervention studies, not addressing any health behaviors, or not conducted in the USA. The full text of 47 relevant articles were reviewed and 9 articles were excluded at this stage due to either health behavior change not being a primary outcome or the study did not including at least one comparison or CG. Finally, 38 articles were identified to meet all of our inclusion criteria and were downloaded into the Endnote electronic reference manager (Figure 1).

Overall, interventions ranged in length from one 60–90 minute session to multiple sessions lasting 24 weeks, with the overall number of sessions lasting on average between 6 and 12 weeks. Most interventions comprise of modules or activity components ranging from 1 to 73 sessions. Below, the researchers discussed review analyses of studies by different targeted health behaviors on the extracted variables described under the methods.

### 3.1. Tobacco prevention

A total of five studies reviewed focused on tobacco use, with sample sizes ranging from 139 to 2523 tobacco users (Patten et al., 2006; Severson, Gordon, Danaher, & Akers, 2008). One intervention targeted adolescents (Patten et al., 2006), whereas the four remaining interventions focused on adult smokers (18+ years old). The most common recruitment methods were flyers, press releases, advertising in newspapers, magazines, and Internet sites, such as Google.com. Only one study utilized emails and electronic newsletters, as it was conducted in a worksite setting (Swartz et al., 2006). Approximately half of the smoking studies used the social cognitive theory (SCT) as a theoretical framework and all the studies were randomized control trials. All studies focused on smoking cessation and/or prevention of relapse, and only one study focused on smokeless tobacco cessation (Severson et al., 2008).

All interventions required multiple access attempts to Internet content. Activities and information were provided using between 5 and 38 modules or sessions (Patten et al., 2006; Swartz et al., 2006). Modules were based primarily on smoking cessation and relapse prevention and provided personal quitting assistance, information on barriers, benefits, and/or strategies and nicotine replacement therapy. Some studies provided individual counseling in addition to Internet use, and one study used a virtual counselor as an “automated approximation of live smoking cessation counselor” (Swartz et al., 2006). The interventions used peer support forums, expert support options, video testimonials, or links to other Internet sites. Most of the interventions reviewed offered personalized messages for participants in order to target a specific behavior. For example, in one study, participants were reminded or encouraged to continue using the intervention even if they did not log on in a week (Japuntich et al., 2006). Some other studies did not permit staff to have any contact with participants during web intervention.

Two studies found higher quit or cessation rates in intervention groups (IGs) than in CGs (Severson et al., 2008; Swartz et al., 2006). Three studies found no significant differences on quit-rates between the IG and CG at the end of the intervention, nor at follow-ups (Japuntich et al., 2006; McKay et al., 2008; Patten et al., 2006).

### 3.2. Alcohol prevention

Four studies reviewed focused on alcohol; all had sample sizes less than 300 participants, with the exception of one that had 1137 (Bingham et al., 2010). All participants were young adults or college aged. Only one college-based study used a convenience sample from college courses (Moore et al., 2005) and the other two recruited primarily via email invitations (Bingham et al., 2010; Neighbors et al., 2009). The one non-college-based study recruited their sample through Human Resource departments in various companies (Doumas & Hannah, 2008). The common goal in all the studies was to reduce alcohol use but each study focused on different aspects. For example, one study focused on binge drinking, whereas another focused on reducing 21st birthday drinking (Moore et al., 2005; Neighbors et al., 2009). Three out of four studies used randomized control designs.

Theoretical frameworks to guide the studies design and methodology included the motivational enhancement model, social norm, and a hybrid model based on four traditional health behavior theories (health belief model [HBM], theory of planned behavior, transtheoretical model [TTM], and precaution process adoption model) (Bingham et al., 2010; Doumas & Hannah, 2008). Different behavior change strategies were used for each intervention. Personalized feedback about drinking behavior was provided in each intervention with the exception of one that used newsletters delivered in print and web versions (Moore et al., 2005). Interactive interventions included online sessions tailored to the participants' alcohol-related risks, allowed participants to print blood alcohol concentration (BAC) chart based on gender and weight, and provided graphed feedback for a visual display of drinking levels related to financial cost (Bingham et al., 2010; Neighbors et al., 2009). All studies reviewed on alcohol Internet interventions demonstrated effectiveness on decreasing drinking in IG, except one (Moore et al., 2005).

### 3.3. Weight loss

A total of seven studies focused on weight loss. Six of the studies had samples ranging between 57 and 192, whereas one study sampled 2862 participants (Rothert et al., 2006). All the studies targeted overweight and/or obese persons except for one that targeted African-American (AA) girls with at least one obese parent and allowed parents to participate in the study (Williamson et al., 2005). Most of the studies recruited participants via newspaper ads, and some included supplemental recruitment strategies, such as community talks, flyers, announcements on websites and listservs, utilization of integrated health care system network for clinician referrals, and letters to members in diabetes and cardiovascular disease (CVD) registries. The primary objectives across all the studies were to reduce weight and behavior modifications.

All the studies followed a randomized controlled trial (RCT) design. However, no specific theoretical framework was identified or used for most studies. The intervention strategies consisted of primarily behavioral modification treatment, mostly via interactive internet counseling, weekly online chats led by trained therapists, and weekly feedback given by either the computer program or tailored messages from a counselor (Gold et al., 2007; Jones et al., 2008; Tate et al., 2006; Webber et al., 2008; Williamson et al., 2005). All the interventions focused on providing a tailored action plan for the participants in the IG. For example, one designed an online therapist-led weight maintenance program (Gold et al., 2007), and another one used email counseling, with all groups meeting at least once for a face-to-face session (Tate et al., 2006). Only one intervention used social media to influence weight loss, using a weight-loss counselor-posted messages daily to reinforce the podcasts and stimulate discussions (Turner-McGrievy & Tate, 2013). When compared to CGs, most of the IGs lost more body fat, body weight, dietary fat intake, and maintained higher weight loss at 12 months (Gold et al., 2007) except for two studies in which the use of an Internet motivation-based intervention did not result in any weight loss (Webber et al., 2008).

### 3.4. PA and nutrition

There were seven studies focused primarily on increasing PA, and additionally five studies also examined related factors, such as nutrition and binge eating. Two studies were categorized as nutrition only interventions, with one focused on folic acid intake and the other targeted fruit juice and vegetable (FJV) consumptions (Milan & White, 2010; Thompson et al., 2009). Sample sizes ranged from 22 to 368 participants in studies targeting PA only (Bosak et al., 2009; Carr et al., 2008; Dunton & Robertson, 2008; Irvine et al., 2013; Marcus et al., 2007; Ornes & Ransdell, 2007). Recruitment methods varied from newspaper advertisements to email solicitations, clinic database selection, and researcher presentations in a variety of settings. In the studies targeting both nutrition and PA, sample sizes were generally larger, ranging from 80 to 1071 participants (Thompson et al., 2008; Winett, Anderson, Wojcik, Winnett, & Bowden, 2007). Recruitment methods were also varied and included emails from management in work settings, voluntary sign-ups in the university setting, and pulpit announcements and bulletins in the church setting (Cook et al., 2007; Franko et al., 2008; Winett et al., 2007). Both types of studies targeted a variety of populations, such as AA girls, university and high school students, and company employees. The studies that examined additional factors related to PA were more likely to target increasing motivation to change health behaviors, increase knowledge, and improve social support and attitudes towards exercise, rather than just an outcome of increased PA and healthier eating. Most of the studies used a RCT design and were based on the SCT. Additional theories guiding the interventions were the TTM and HBM.

Most of the intervention strategies to increase primarily PA included behavior-specific goal-setting approaches, and all the interventions allowed participants to monitor or record their PA progress and receive tailored individualized feedback. Despite fairly similar intervention strategies were used, information was disseminated in a variety of ways. One study provided new content on the website each week including links for evidence-based websites (Bosak et al., 2009), whereas another administered weekly emails with links to interactive tailored web pages to the IG (Dunton & Robertson, 2008), and yet another study delivered the intervention in four weeks through nine modules via WebCT (Ornes & Ransdell, 2007), the final study included a social media campaign using Facebook and the Internet Support for Health Associations Promoting Exercising (INSHAPE) to encourage participation from the participants (Cavallo et al., 2012).

Likewise, the studies focusing on additional factors related to PA also included goal setting as a key feature of the intervention programs, allowed the participants to self-monitor their progress and report it for feedback. Most modules and new topics were introduced weekly, and interactive components included group discussions, assessment pages, resources, and motivational messages. Innovative strategies that were not common in all the groups included the use of comics and role modeling on the intervention website, and the provision of motivational support from the church pulpit announcement and bulletins (Thompson et al., 2008, 2009; Winett et al., 2007).

All the interventions were successful in the studies focusing primarily on PA, except one (Marcus et al., 2007). PA was increased in three IGs, in addition to decreased waist circumference and coronary risk ratio (Carr et al., 2008). The cost of development and delivery of Internet health interventions was also found to be lower than consultation and follow-up in the clinic (Carr et al., 2008). All the studies examining nutrition alone, nutrition and other factors in addition to PA reported increases in healthy dietary behaviors and PA levels in the IGs; except for one study that found no differences on PA or stress (Cook et al., 2007).

### 3.5. HIV and chronic diseases

Four studies focused on HIV prevention or sexual health, and four studies focused on other chronic diseases: diabetes management, colon cancer screening, asthma management, and depression in stroke survivors and their caregivers (Bond et al., 2007; Chan & Vernon, 2008; Joseph et al., 2007; Smith et al., 2012). The HIV prevention studies recruited participants online, in addition to clinic-based referrals (Bull et al., 2008) and face-to-face recruitment (Bowen et al., 2007). Two studies targeted men who have sex with other men (MSMs) and enrolled a maximum of 112 participants (Bowen et al., 2007; Carpenter et al., 2009), whereas one targeted youth regardless of sexual orientation and enrolled approximately 4000 in their study (Bull et al., 2008). For the other chronic disease studies, the number of participants did not exceed 408.

Most of the studies were RCTs. HIV prevention studies used a combination of strategies based on constructs from primarily three theoretical frameworks: the SCT, information–motivation–behavioral skills model, and motivational interviewing (MI). Among the chronic disease studies, three did not use any particular theoretical framework while one used both the TTM and HBM (Joseph et al., 2007).

HIV prevention studies used a range of 2–7 modules; these were tailored for the specific population and included questions related to HIV risk, pictures and audio role model stories, interactive graphic conversations between two men of different HIV status, and motivation, information and, skills training (Bowen et al., 2007; Bull et al., 2008; Carpenter et al., 2009). Only one study mentioned providing tailored feedback throughout the intervention (Carpenter et al., 2009). The sexual health intervention focused on consistent condom use using Facebook to deliver information to the AA and Latino youth. Results showed condom use remained stable in the IG and declined in the CG (Bull et al., 2012).

The diabetes management intervention focused its strategies primarily on goal-setting and problem-solving skills (Bond et al., 2007). The asthma management study used approximately five tailored educational modules to make use of feedback from previous sessions and correspond to participants' stage of readiness (Joseph et al., 2007). The intervention focused on colon cancer screening study was the only one to include involvement from a primary care physician and record biomarkers (Chan & Vernon, 2008). The intervention on depression management in stroke survivors and their caregivers provided caregivers with knowledge, resources, and skills to reduce distress and provide best care to their stroke survivors (Smith et al., 2012).

Most of these Internet health interventions demonstrated significant effectiveness in increased HIV/AIDS knowledge and self-efficacy, decreased number of unprotected sexual acts, reduced HbA1c, weight and cholesterol levels, and fewer asthma symptoms and related hospitalizations (Bond et al., 2007; Bowen et al., 2007; Joseph et al., 2007; Lustria, Cortese, Noar, & Glueckauf, 2009). Only one study was considered ineffective as the public access arm of the study yielded a higher return rate of the fecal occult blood test (FOBT) for colon screening from CG than IG participants (33%) (Chan & Vernon, 2008).

### 3.6. Social media

Of the articles reviewed, only three included social media interventions; two used Facebook and one used Twitter. For each of these studies, the researchers use a trained professional that facilitated discussions on the social media outlet. Cavallo et al. (2012) used Facebook and a program called INSHAPE to encourage PA in college students. Bull et al. (2012) used Facebook to facilitate consistent condom use in teenagers in California; and Turner-McGrievy and Tate (2013) used Twitter and podcast to promote weight loss. The moderators in these campaigns were trained counselors for the specific health behavior that the program focused on.

## 4. Discussion

Findings from the current review study indicated that, overall, Internet or web-based interventions (WIs) produce favorable results and are effective in producing and increasing targeted health or behavioral outcomes. Common trends for particular methods to recruit participants in the Internet health intervention studies throughout the review typically included some form of media, such as newspaper advertisements and announcements, press releases, television advertisements, posters and flyers, and internet-based recruitment (banners and/or paid advertisements on websites). Existing successful studies have commonly used a combination of methods for recruitment instead of relying solely on one method, clinical referrals for instance. The review indicates that specific settings and/or target populations use certain recruitment strategies more often. For example, school-based interventions typically recruit in classes and school clinics used flyers, emails, and student rallies, whereas those based in the community are more inclined to use newspaper, radio and television media, billboards, flyers, and even pulpit announcements. Financial incentives were most often used as a strategy to increase retention, and they ranged from $5 to raffle prizes of $500. Most studies offered $10–30 to participate in either each session or at the completion of the study, as an incentive.

Intervention time length and number of modules do not appear to be highly correlated with the success rate of interventions; i.e. shorter interventions or those with fewer modules do not necessarily result in less desirable outcome measures than long interventions or those with more modules. Instead, intervention components, such as modules, structure, and interaction nature of the intervention, seemed having a greater impact on the measured outcomes. For instance, it was evident that the involvement of professional or a trained counselor to provide professional advice during online interventions was often associated with positive significant intervention effects on the target population, especially in the case of smoking cessation, weight loss, and social media-related interventions. This is not surprising as the interaction of experts with participants is expected to be more substantial through the provision of direct feedback and monitoring (Dijkstra & de Vries, 1999).

Common strategies identified, including the use of web-based information, tailored feedback, weekly emails, goal setting, and self-assessment. A handful of studies also used innovative intervention strategies, such as the use of familiar/relatable avatars, humor in a birthday card message, individual graphed feedback and self-directed programs. Our review consistently showed that Internet health interventions using a combination of approaches or methods often resulted in effective intervention outcomes.

The SCT and the TTM were the most commonly used theoretical foundations for all the interventions, particularly within the smoking cessation and PA/nutrition intervention studies, with the SCT used in more than half of the studies. Most of the interventions are grounded in theoretical frameworks, except that the majority of the weight-loss interventions did not identify specific theoretical frameworks. Much research underscores the importance of using theory as a basis for behavioral interventions. Theoretical models such as the HBM, Theory of Planned Behavior, and SCT are among those most often used in health education, therefore serve as an excellent frame of reference for Internet health interventions. Our review supports the use and application of theories as a foundation for behavior change interventions would be expected to increase the likelihood of effectiveness for the intervention (Hamel et al., 2011). Additionally, the literature on Internet health interventions indicates that the use of behavioral theories could facilitate program exposure (Crutzen et al., 2011). Although most of the successful alcohol internet intervention studies reviewed did not specify theoretical frameworks, majority of these studies did indicate using tailored messages or tailored action plan, and/or Internet counseling/chats led by trained therapists. In one systematic review of computer-tailored behavioral interventions, researchers also noted that the utilization of tailoring based on population characteristics, health foci, behaviors, and situations are necessary to maximizing the effectiveness of computer-assisted tailored programs (Risk & Dzenowagis, 2001).

All in all, the majority of interventions reviewed yielded significant improvement on health behavioral outcomes. This review indicates that using multi-dimensional approaches, such as tailored intervention components and personalized feedback, is associated with favorable intervention effects. Almost all the interventions across health issues showed significant impact on health outcomes, except the tobacco Internet health interventions. Most of the tobacco Internet interventions yielded non-significant results for cessation and quit-rates, although the results were mostly noted as promising. It is not clear why these interventions did not show effectiveness. Some recommendations proposed are that longer follow-up periods be implemented to determine long-term impacts of programs, and that sample sizes be increased to increase statistical power to possibly detect any long-term significant differences (Patten et al., 2006; Swartz et al., 2006).

This review study has a few limitations. The relationship between the components of the intervention and actual outcome measured although associated may not be causative. Our review was limited to only interventions delivered to target groups in the USA and may not be representative of the acceptability and utility of internet health interventions overall worldwide. To make the scope of this review manageable, two major electronic databases were searched to retrieve published Internet health intervention studies (from 2005 to 2013); i.e. CINAHL and Medline; thus our results and analyses should not be considered an exhaustive analysis. Furthermore, our review might suffer risk of bias commonly exists in systematic review studies, including the publication bias, selective reporting within studies, and/or incomplete retrieval of identified research and reporting bias. Finally, due to the diversity of the type of programs as well as the process and/or outcome variables used, it was unfeasible to conduct meaningful meta-analyses. For studies that examined some similar health conditions, not all studies examined or analyzed the same outcome variables. Although it would also be nice to be able to analyze different population groups, many studies had mixed age or gender participants. In addition, existing Internet intervention programs reviewed commonly used multiple strategies and generally did not examine or provide sufficient information on the effectiveness of specific strategies. Our review provided thoughtful narrative analyses on some of these areas the best we can, given available data and information provided in existing studies.

Nevertheless, this review clearly shows that a broad range of successful Internet health interventions has been conducted and they all share some common themes across their topics related to their success. The researchers conducting this review made every effort to include all relevant evidence pertaining to this topic published in English in the USA. The comprehensiveness of this review allows a broader review scope and an enhanced ability to consider and discuss themes as they occurred across various health conditions and behaviors. It serves as an important update on the rapid progress of interventions developed in the recent years and provides synthesized knowledge on the design and effectiveness of delivering Internet health interventions.

Overall, the results lend support to the use of the Internet or Web as a viable alternative to more traditional health interventions as a feasible means for health promotion and disease prevention. Health practitioners are encouraged to consider incorporating the use of technology into more traditional intervention approaches to enhance the overall outcomes among their target population. Further research is needed to evaluate participants' satisfaction with the Internet health interventions compared to more traditional approaches. Additionally, future studies are encouraged to examine what dose or exposure to Internet health interventions is optimum to achieve outcomes across various health behaviors or conditions.

## References

[CIT0001] Bennett G. V., Galsgow R. E. (2009). The delivery of public health interventions via the internet: Actualizing their potential. *Annual Review of Public Health*.

[CIT0002] Bingham C. R., AI Barretto M. A., Walton C. M., Bryant J., Shope T., Raghunathan T. E. (2010). Efficacy of a web-based, tailored, alcohol prevention/intervention program for college students: Initial findings. *Journal of American College Health*.

[CIT0003] Bond G. E., Burr R., Wolf F. M., Price M., McCurry S. M., Teri L. (2007). The effects of a web-based intervention on the physical outcomes associated with diabetes among adults aged 60 and older: A randomized trial. *Diabetes Technology and Therapeutics*.

[CIT0004] Bosak K. A., Tyates B., Pozehl B. (2009). Feasibility of an internet physical activity intervention. *Western Journal of Nursing Research*.

[CIT0005] Bowen A. M., Keith H., Mark L. W. (2007). A randomized control trial of internet-delivered HIV prevention targeting rural MSM. *Health Education Research*.

[CIT0006] Bull S. S., Levine D. K., Black S. R., Schmiege S. J., Santelli J. (2012). Social media-delivered sexual health intervention: A cluster randomized controlled trial. *American Journal of Preventative Medicine*.

[CIT0007] Bull S. S., Vallejos D., Levine D. K., Ortiz C. (2008). Improving recruitment and retention for an online randomized controlled trial: Experience from the youthnet study. *AIDS Care*.

[CIT0008] Carpenter K. M., Stoner S. A., Mikko A. N., Dhanak L. P.,, Parsons J. T. (2009). Efficacy of a web-based intervention to reduce sexual risk in men who have sex with men. *AIDS and Behavior*.

[CIT0009] Carr L. J., Bartee R. T., Dorozynski C., Broonfield J. F., Smith M. L., Smith D. T. (2008). Internet-delivered behavior change program increases physical activity and improves cardiometabolic disease risk factors in sedentary adults: Results of a randomized controlled trial. *Preventive Medicine*.

[CIT0010] Cavallo D. N., Tate D. F., Ries A. V., Brown J. D., DeVellis R. F., Ammerman A. S. (2012). A social media-based physical activity intervention: A randomized controlled trial. *American Journal of Preventative Medicine*.

[CIT0011] Chan E. C. Y., Vernon S. W. (2008). Implementing an intervention to promote colon cancer screening through e-mail over the internet: Lessons learned from a pilot study. *Medical Care*.

[CIT0012] Cook R. F., Billings D. W., Hersch R. K., Back A. S., Hendrickson A. (2007). A field test of a web-based workplace health promotion program to improve dietary practices, reduce stress, and increase physical activity: Randomized controlled trial. *Journal of Medical Internet Research*.

[CIT0013] Crutzen R., de Nooijer J., Brouwer W., Oenema A., Brug J., de Vries N. K. (2011). Strategies to facilitate exposure to internet-delivered health behavior change interventions aimed at adolescents or young adults: A systematic review. *Health Education & Behavior*.

[CIT0014] Dijkstra A., de Vries H. (1999). The development of computer-generated tailored interventions. *Patient Education and Counseling*.

[CIT0015] Doumas D. M., Hannah E. (2008). Prevention high-risk drinking in youth in the workplace: A web-based normative feedback program. *Journal of Substance Abuse Treatment*.

[CIT0016] Dunton G. F., Robertson T. P. (2008). A tailored internet-plus-email intervention for increasing physical activity among ethnically-diverse women. *Preventive Medicine*.

[CIT0017] Frenn, M., Malin, S., Brown, R. L., Greer, Y., Fox, J., Breer, J., & Smyczek, S. (2005). Changing the tide: An Internet/video exercise and low-fat diet intervention with middle-school students. *Applied Nursing Research, 18*(1), 13–21. 10.1016/j.apnr.2004.04.003 15812731

[CIT0018] Franko D. L., Cousineau T. M., Trant M., Green T. C., Rancourt D., Thompson D., … Ciccazzo M. (2008). Motivation, self-efficacy, physical activity and nutrition in college students: Randomized controlled trial of an internet-based education program.

[CIT0019] Gold B. C., Burke S., Pintauro S., Buzzell P., Harvey-Perino J. (2007). Weight loss on the web: A pilot study comparing a structured behavioral intervention to a commercial program. *Obesity (Silver Spring, MD)*.

[CIT0020] Hamel L. M., Robbins L. B., Wilbur J. (2011). Computer- and web-based interventions to increase preadolescent and adolescent physical activity: A systematic review. *Journal of Advances Nursing*.

[CIT0021] Irvine B. A., Gelatt V. A., Seeley J. R., Macfarlane P., Gau J. M. (2013). Web-based intervention to promote physical activity by sedentary older adults: Randomized controlled trial. *Journal of Medical Internet Research*.

[CIT0022] Japuntich S. J., Zehner M. E., Smith S. S., Jorenby D. E., Valdez J. A., Fiore M. C., … Gustafson D. H. (2006). Smoking cessation via the internet: A randomized clinical trial of an internet intervention as adjuvant treatment in a smoking cessation Intervention. *Nicotine and Tobacco Research: Official Journal of the Society for Research on Nicotine and Tobacco*.

[CIT0023] Jones M., Luce K. H., Osborne M. I., Taylor K., Cunning D., Doyle A. C., … Taylor C. B. (2008). Randomized, controlled trail of an internet-facilitated intervention for reducing binge eating and overweight in adolescents. *Pediatrics*.

[CIT0024] Joseph C. L. M., Peterson E., Havstad S., Johnson S. S., Hoerauf S., Stringer S., … Stretcher V. (2007). A web-based, tailored asthma management program for urban African-American high school students. *American Journal of Respiratory and Critical Care Medicine*.

[CIT0025] Lustria M. L. A., Cortese J., Noar S. M., Glueckauf R. L. (2009). Computer-tailored health interventions delivered over the web: Review and analysis of key components. *Patient Education and Counseling*.

[CIT0026] Marcus B. H., Lewis B. A., Williams D. M., Dunsiger S., Jackicic J. M., Whiteley J. A., … Parisi A. F. (2007). A comparison of internet and print-based physical activity interventions. *Archives of Internal Medicine*.

[CIT0027] McKay H. G., Danaher B. G., Seeley J. R., Lichtenstein E., Gau J. M. (2008). Comparing two web-based smoking cessation programs: Randomized controlled trial. *Journal of Medical Internet Research*.

[CIT0028] Milan J. E., White A. A. (2010). Impact of a stage-tailored, web-based intervention on folic acid-containing multivitamin use by college students. *American Journal of Health Promotion*.

[CIT0029] Moore M. J., Soderquist J., Werch C. (2005). Feasibility and efficacy of a binge drinking prevention intervention for college students delivered via the internet versus postal mail. *Journal of American College Health*.

[CIT0030] Neighbors C., Lee C. M., Lewis M. A., Fossos N., Walter T. (2009). Internet-based personalized feedback to reduce 21st-birthday drinking: A randomized controlled trail of an event-specific prevention intervention. *Journal of Consulting and Clinical Psychology*.

[CIT0031] Neuhauser L., Kreps G. L. (2010). eHealth communication and behavior change: Promise and performance. *Social Semiotics*.

[CIT0032] Ornes L., Ransdell L. B. (2007). Web-based physical activity intervention for college-aged women. *International Electronic Journal of Health Education*.

[CIT0033] Patten C. A., Croghan I. T., Meis T. M., Decker P. A., Pingree S., Colligan R. C., … Baumberger R. K. (2006). Randomized clinical trial of an internet-based versus brief office intervention for adolescent smoking cessation. *Patient Education and Counseling*.

[CIT0034] http://dx.doi.org/10.2196/jmir.3.4.e28.

[CIT0035] Rothert K., Stretcher V. J., Doyle L. A., Caplan W. M., Joyce J. S., Jimison H. B., … Roth M. A. (2006). Web-based weight management programs in an integrated health care setting: A randomized, controlled trial. *Obesity (Silver Spring, MD)*.

[CIT0036] Severson H. H., Gordon J. S., Danaher B. G., Akers L. (2008). Chewfree.com: Evaluation of a web-based cessation program for smokeless tobacco users. *Nicotine and Tobacco Research: Official Journal of the Society for Research on Nicotine and Tobacco*.

[CIT0037] Smith G. G., Egbert N., Dellman-Jenkins M., Nanna K. (2012). Reducing depression in stroke survivors and their informal caregivers: A randomized clinical trial of a web-based intervention. *Rehabilitation Psychology*.

[CIT0038] Swartz L. H., Noell J. W., Schroeder S. W., Ary D. V. (2006). A randomised control study of a fully automated internet based smoking cessation programme. *Tobacco Control*.

[CIT0039] Tate D. F., Jackvony E. H., Wing R. R. (2006). A randomized trial comparing human e-mail counseling, computer-automated tailored counseling, and no counseling in an internet weight loss program. *Archives of Internal Medicine*.

[CIT0040] Thompson D., Baranowski T., Baranowski J., Cullen K., Jago R., Watson K., Liu K. (2009). Boy scout 5-a-day badge: Outcome results of a troop and internet intervention. *Preventive Medicine*.

[CIT0041] Thompson D., Baranowski T., Cullen K., Watson K., Liu Y., Canada A., … Zakeri I. (2008). Food, fun, and fitness internet program for girls: Pilot evaluation of an e-health youth obesity prevention program examining predictors of obesity. *Preventive Medicine*.

[CIT0042] Turner-McGrievy G. M., Tate D. F. (2013).

[CIT0043] Webber K. H., Tate D. F., Bowling M. (2008). A randomize comparison of two motivationally enhanced internet behavioral weight loss programs. *Behaviour Research and Therapy*.

[CIT0044] Williamson D. A., Martin P. D., White M., Newton R., Walden H., York-Crowe E., … Ryan D. (2005). Efficacy of an internet-based behavioral weight loss program for overweight adolescent African-American girls. *Eating and Weight Disorders*.

[CIT0045] Winett R. A., Anderson E. S., Wojcik J. R., Winnett S. G., Bowden T. (2007). Guide to health: Nutrition and physical activity outcomes of a group-randomized trial of an internet-based intervention in churches. *Annals of Behavioral Medicine: A Publication of the Society of Behavioral Medicine*.

